# The association between non-high-density lipoprotein cholesterol to high-density lipoprotein cholesterol ratio and pulmonary function: evidence from NHANES 2007–2012

**DOI:** 10.3389/fnut.2025.1534958

**Published:** 2025-03-19

**Authors:** Miaoyan Liu, Chaofeng Gao, Jinggeng Li, Yibo Zhang, Rui Gao, Chaoting Yang, Jian Zhang

**Affiliations:** ^1^Department of Respiratory Medicine, Chest Hospital, Xi'an People's Hospital (Xi'an Fourth Hospital), Xi'an, China; ^2^Department of General Surgery, Lanzhou University Second Hospital, Lanzhou, China; ^3^Department of Respiratory and Critical Care Medicine, First Affiliated Hospital of Air Force Medical University, Xi'an, China; ^4^Graduate Work Department, Xi'an Medical University, Xi'an, China

**Keywords:** NHHR, pulmonary function, NHANES, lipids, cross-sectional research

## Abstract

**Background:**

This research aims to explore the potential association between lung function and the ratio of non-high-density lipoprotein cholesterol (NHL) to high-density lipoprotein cholesterol (NHHR). Previous research has shown that lipid metabolism imbalance is closely linked to cardiovascular disease, however, there is a lack of information regarding its impact on lung function.

**Methods:**

This research used information from the National Health and Nutrition Examination Survey (NHANES) spanning the years 2007 to 2012, including a large-scale sample of 9,498 adults aged 20 years and older. A cross-sectional study employing multivariable regression models was aimed at examining the relevance between NHHR and indicators of lung function (FEV1, FVC, and FEV1/FVC). Adjustments were made for a wide range of confounding factors, encompassing race, gender, age, BMI, smoking status, physical activity, diabetes, alcohol consumption, and education level. Data analysis included categorizing NHHR into quartiles and using trend tests to evaluate dose–response relationships between NHHR quartiles and lung function. Sensitivity analyses were conducted by excluding participants with asthma and COPD to ensure the reliability of the results.

**Results:**

The results manifested a significant correlation between decreased FEV1 and FVC values and elevated NHHR, most notably within the highest quartile of NHHR (Q4), where the association was most pronounced. Additionally, trend test results indicated a significant linear negative correlation between NHHR and both FEV1 and FVC. However, the correlation between FEV1/FVC and NHHR showed a nonlinear U-shaped pattern. Suggesting differential impacts of NHHR on various lung function indicators. The findings’ robustness was shown by sensitivity analysis, which revealed that even after omitting people with asthma and COPD, the negative correlation between NHHR and FEV1 and FVC remained significant.

**Conclusion:**

This research emphasizes the significance of tracking lipid levels in evaluating respiratory health and offers early evidence in favor of NHHR as a probable biomarker for respiratory function. Further longitudinal research has occasion to prove the causal relationship between NHHR and lung function and to explore its underlying biological mechanisms.

## 1 Introduction

Pulmonary ventilation and gas exchange are critical components of the respiratory process, ensuring the body can efficiently obtain oxygen and expel carbon dioxide (1). Pulmonary function tests (PFTs) are non-invasive physical evaluation methods that provide valuable insights into lung health (2). Standard clinical measures of pulmonary function comprise forced expiratory volume in 1 s (FEV1), the forced vital capacity (FVC), as well as the ratio of FEV1 to FVC (3). These metrics are essential for diagnosing and assessing a wide range of pulmonary diseases.

The findings of recent research, lipid levels and metabolic health may potentially have an impact on lung function in addition to the respiratory system (4, 5). Dyslipidemia, which refers to an imbalance in blood lipid levels—Usually defined by either decreased HDL-C or increased LDL-C, this condition is directly linked to a number of negative health outcomes, particularly atherosclerosis, coronary disease, myocardial infarction, and hypertension (6–9). Additionally, dyslipidemia is significantly linked to metabolic disorders and may even increase the chance of developing renal disorders and cancer (10–12). However, the link between dyslipidemia and PFT not fully explored yet.

The non-HDL-C/HDL-C (NHHR), represents the proportion of non-high-density lipoprotein cholesterol (non-HDL-C) relative to high-density lipoprotein cholesterol (HDL-C) and serves as a plausible indicator of lipid dysregulation. According to recent studies, NHHR is extensively linked to a number of disorders, including one by Wu et al. (13, 14), they found very substantial association between chronic obstructive pulmonary disease (COPD) and NHHR, with the Dietary Inflammatory Index playing a mediating role (15). However, little investigation has been done on the connection between NHHR and assess lung function among the general populace. Therefore, the intent of this study is to further explore the potential association between NHHR and lung function using data from NHANES.

## 2 Methods

### 2.1 Study population

**Figure 1:**
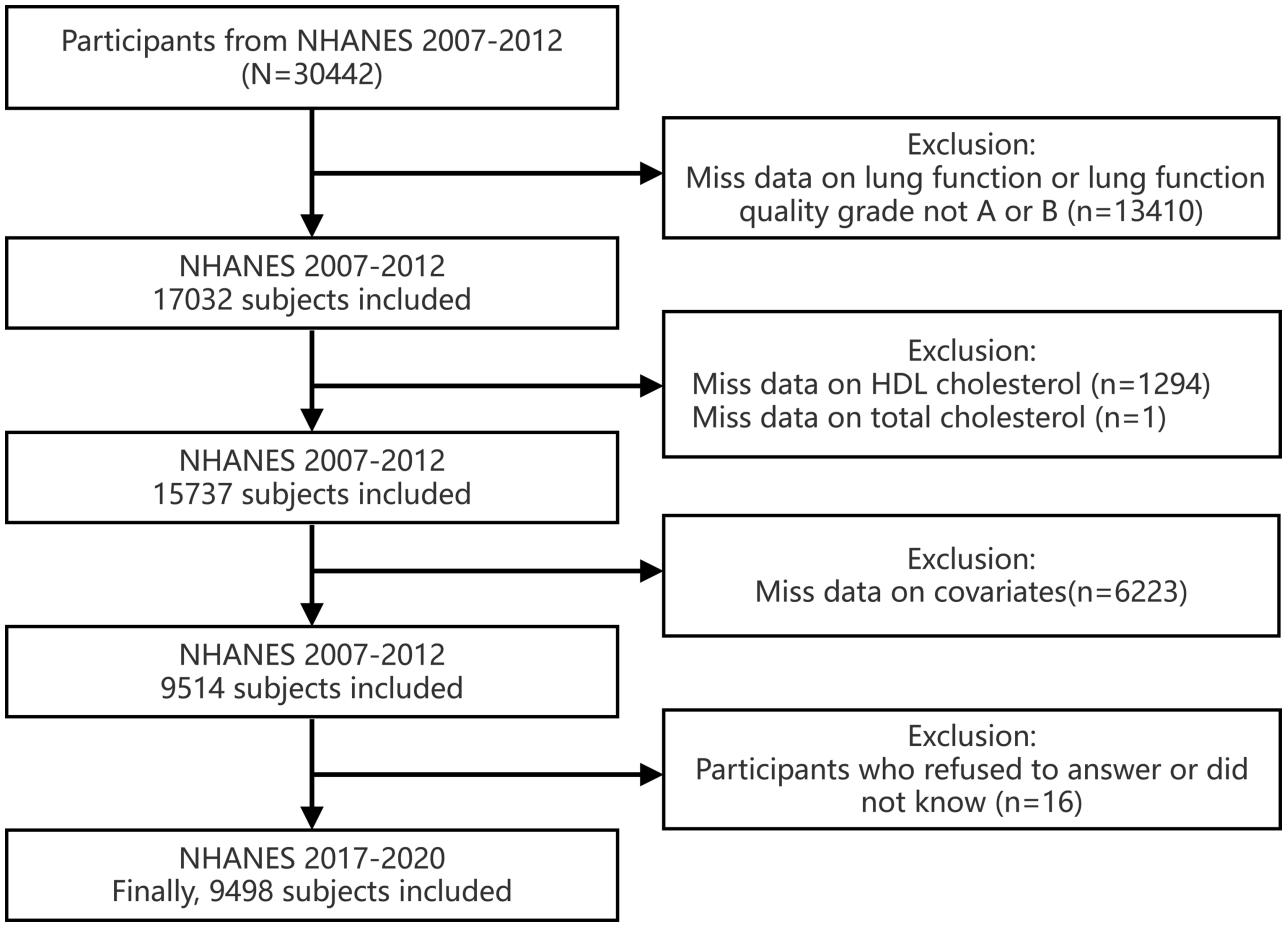
Flowchart of study cohort selection process.

This research utilized a population-based retrospective and cross-sectional design to analyze data from the NHANES for the years 2007–2012. NHANES employs an integrated multi-stage sampling strategy to ensure national representativeness, assessing individual nourishment and health condition throughout America communities. Participants underwent thorough evaluations, this encompasses household interviews and assessments conducted at Mobile Examination Centers (MECs). Exclusion criteria were applied to participants as follows: (1) those missing pulmonary function data, including FEV1 and FVC, or those whose pulmonary function test quality was graded below A or B; (2) those missing high-density lipoprotein cholesterol or total cholesterol information; (3) individuals lack covariate data; and (4) people who refused to answer or responded “do not know.” Since the covariate of education level was collected only from participants aged 20 and above, this research exclusively involved participants who were aged 20 years or above. Ultimately, the analysis comprised 9,498 participants. Figure 1 outlines the detailed screening process.

The Research Ethics Review Board of the National Center for Health Statistics (NCHS) approved the NHANES study protocol, and all participants furnished written informed consent. Consequently, additional ethical approval or consent from participants was deemed unnecessary for the subsequent data analysis.

### 2.2 Assessment of NHHR and pulmonary function

We used the NHHR to evaluate its impact on pulmonary function. The NHHR was calculated using the formula: NHHR = Non-HDL-C/HDL-C, where Non-HDL-C was derived by subtracting HDL-C from total cholesterol (TC) (16). These measurements were obtained through the Mobile Examination Center (MEC). The NHANES Lab Manual provides detailed explanations of the methods for assessing HDL-C and TC. Pulmonary function was assessed using FEV1, FVC, and FEV1/FVC, with measurements conducted by trained health technicians. Each participant’s results were graded for quality, with ratings assigned as A, B, C, D, or F. So as to maintain data integrity, our analysis was restricted to participants who received quality ratings of A and B.

### 2.3 Covariates

Based on clinical insights and previous research (17, 18), we used the following covariates: Age, sex, racial background, body mass index (BMI), serum cotinine levels, educational attainment, poverty income ratio (PIR), levels of physical activity (PA), smoking habits, alcohol intake, diabetes mellitus status, and hypertensive condition are all factors to consider. Serum cotinine, a metabolite of nicotine, was used to reflect smoke exposure and nicotine intake. Supplementary Table S1 provides a detailed elucidation of the classifications and the respective variables involved. All data were processed using the weighting method prescribed by NHANES.

### 2.4 Statistical analysis

According to the guidelines from the official NHANES website, a complex sampling design and corresponding sample weights were employed to produce nationally representative estimates. Continuous variables expressed as weighted means ± standard error (SE), whereas categorical variables were presented as unweighted counts along with their corresponding weighted percentages. To compare the fundamental attributes between NHHR quartiles, for continuous variables, a t-test with weights was utilized, whereas differences in categorical variables were assessed using a weighted chi-square test. The presence of multicollinearity between variables was tested using the Generalized Variance Inflation Factor (GVIF) and Spearman correlation analysis. To evaluate the association between NHHR and pulmonary function indicators (FEV1, FVC, and FEV1/FVC), multivariable linear regression models were utilized. Three distinct models were developed: Model 1 was unadjusted; Model 2 was adjusted for age, gender, and race; and Model 3 was adjusted for all relevant covariates. The results of each model were presented as regression coefficients (*β*) with a confidence interval of 95%(CI). For a more detailed analysis, NHHR was divided into quartiles, and conducted trend testing to evaluate the dose–response relationship between NHHR quartiles and pulmonary function. Since the original values of FEV1/FVC were too small, impacting the interpretation of results, we multiplied FEV1/FVC by 1,000 before conducting multivariable linear regression and subgroup analyses. This adjustment did not alter the direction or significance of the results but allowed for a clearer interpretation of the regression coefficients and confidence intervals. To evaluate non-linear relationships, smoothed curve fitting was performed using generalized additive models (GAM). Furthermore, a threshold effect analysis was executed utilizing two-stage linear regression model to ascertain potential turning points. Subgroup analyses and interaction tests were performed to examine the moderating effects of categorical variables. Sensitivity analyses were performed by omitting participants diagnosed with asthma and COPD to ensure the stability of the results. Statistical significance was established with a threshold of a *p*-value below 0.05. All data analyses were conducted utilizing R Studio (version 4.3.3) and Empower software (version 4.2).

## 3 Results

### 3.1 Baseline characteristics

This research ultimately encompassed 9,498 participants, with an average age of 44.85 ± 15.17 years. Of these, 49% were male and 51% were female, with 67% being Non-Hispanic White. The mean value of FEV1 was 3,242.97 ± 891.66 ml, the mean FVC was 4,159.29 ± 1,077.68 ml, and the mean FEV1/FVC ratio was 0.78 ± 0.08. The participants were divided into quartiles based on their NHHR measurements. Notable variations were identified among the four groups in age, gender, ethnic group, educational level, BMI, physical activity, smoking, diabetes, hypertension, Poverty-Income Ratio (PIR), FEV1 and FVC (*p* < 0.05) (Table 1).

**Table 1:** Study population characteristics.

Characteristic	Overall (0.48–21)	NHHR				*p*-value^2^
		Q1 (0.48–2.03)	Q2 (2.03–2.79)	Q3 (2.79–3.78)	Q4 (3.78–21)	
*N*	9,498 (100%)	2,375 (25.5%)	2,375 (24.9%)	2,374 (25.1%)	2,374 (24.5%)	
Age (years)	44.85 ± 15.17	43.59 ± 16.56	45.12 ± 15.55	45.26 ± 14.78	45.47 ± 13.49	0.006
Gender						<0.001
Female	4,796 (51%)	1,545 (68%)	1,342 (58%)	1,059 (44%)	850 (35%)	
Male	4,702 (49%)	830 (32%)	1,033 (42%)	1,315 (56%)	1,524 (65%)	
Race						<0.001
Mexican American	1,476 (7.8%)	246 (5.4%)	331 (6.9%)	420 (9.0%)	479 (10%)	
Other Hispanic	974 (5.1%)	192 (4.0%)	241 (4.7%)	282 (6.1%)	259 (5.5%)	
Non-Hispanic White	4,464 (72%)	1,122 (72%)	1,106 (72%)	1,068 (70%)	1,168 (72%)	
Non-Hispanic Black	1853 (9.5%)	611 (13%)	503 (10%)	421 (8.7%)	318 (6.6%)	
Other race	731 (5.9%)	204 (5.8%)	194 (5.9%)	183 (6.4%)	150 (5.5%)	
Education level						<0.001
≤High school	4,304 (37%)	887 (29%)	1,013 (34%)	1,145 (40%)	1,259 (45%)	
>High school	5,194 (63%)	1,488 (71%)	1,362 (66%)	1,229 (60%)	1,115 (55%)	
BMI group						<0.001
<25	2,703 (31%)	1,194 (54%)	737 (34%)	465 (21%)	307 (13%)	
25–30	3,191 (34%)	685 (28%)	805 (35%)	864 (37%)	837 (36%)	
≥30	3,604 (35%)	496 (17%)	833 (32%)	1,045 (42%)	1,230 (52%)	
Physical activity						<0.001
Yes	4,907 (58%)	1,366 (63%)	1,253 (60%)	1,194 (56%)	1,094 (52%)	
No	4,591 (42%)	1,009 (37%)	1,122 (40%)	1,180 (44%)	1,280 (48%)	
Smoking status						<0.001
Never	5,146 (55%)	1,417 (58%)	1,329 (56%)	1,275 (55%)	1,125 (50%)	
Former	2,223 (24%)	506 (24%)	579 (26%)	604 (26%)	534 (22%)	
Current	2,129 (21%)	452 (18%)	467 (18%)	495 (20%)	715 (28%)	
Diabetes						<0.001
Yes	1,333 (10%)	249 (6.8%)	319 (8.9%)	350 (11%)	415 (13%)	
No	8,165 (90%)	2,126 (93%)	2056 (91%)	2024 (89%)	1959 (87%)	
Alcohol						0.3
Yes	7,238 (81%)	1814 (81%)	1780 (81%)	1786 (80%)	1858 (83%)	
No	2,260 (19%)	561 (19%)	595 (19%)	588 (20%)	516 (17%)	
Hypertension						<0.001
No	6,535 (72%)	1731 (78%)	1,653 (74%)	1,585 (70%)	1,566 (67%)	
Yes	2,963 (28%)	644 (22%)	722 (26%)	789 (30%)	808 (33%)	
PIR	3.13 ± 1.65	3.22 ± 1.67	3.20 ± 1.64	3.13 ± 1.65	2.98 ± 1.64	0.002
FEV1	3,242.97 ± 891.66	3,154.34 ± 861.66	3,191.78 ± 902.92	3,290.32 ± 914.30	3,338.89 ± 875.25	<0.001
FVC	4,159.29 ± 1,077.68	4,009.00 ± 1,012.34	4,098.55 ± 1,081.68	4,234.53 ± 1,117.60	4,300.61 ± 1,074.00	<0.001
FEV1/FVC	0.78 ± 0.08	0.79 ± 0.08	0.78 ± 0.08	0.78 ± 0.08	0.78 ± 0.07	0.060

^1^Mean ± SD for continuous; *n* (unweighted) (%) for categorical. ^2^Wilcoxon rank-sum test for complex survey samples; chi-squared test with Rao & Scott’s second-order correction. ^3^BMI: Body Mass Index, PIR: Poverty Income Ratio, FEV1: Forced Expiratory Volume in 1 s, FVC: Forced Vital Capacity, FEV1/FVC: Forced Expiratory Volume in 1 s/ Forced Vital Capacity.

### 3.2 The relationship between NHHR and pulmonary function

To ensure the robustness of the model, we assessed the multicollinearity and correlations among variables. The calculated Generalized Variance Inflation Factor (GVIF) values (Supplementary Table S2) were all below 10, indicating no significant multicollinearity. The Spearman correlation heatmap (Supplementary Figure S1) showed that most variables had weak correlations (|*ρ*| < 0.5), suggesting minimal overlap among them. To achieve the objective of assessing the association between NHHR and pulmonary function, we used three multivariable regression models, as shown in Table 2. The findings indicated a noteworthy correlation between NHHR and pulmonary function. In Model 1, without taking into account any confounding variables, the NHHR positively correlated with both FEV1 and FVC, while demonstrating a negative correlation with the FEV1/FVC ratio. In Model 2, subsequent to the adjustment for variables such as age, gender, and race. The NHHR demonstrated a significant inverse relationship with both FEV1 and FVC, while no significant relationship was observed with the FEV1/FVC ratio. In Model 3, subsequent to the adjustment for all covariates, NHHR exhibited a substantial negative correlation with FEV1 (*β*: −15, 95% CI: −28, −2.1) and FVC (β: −21, 95% CI: −35, −7.2), with no significant association with FEV1/FVC. To investigate the intricate correlation between NHHR and pulmonary function in greater depth, NHHR was categorized into quartiles for the purpose of analysis. The results indicated that in Model 3, higher NHHR quartiles (especially Q4) were more significantly negatively associated with FEV1 (Model 3: *β* for Q4: −60, 95% CI: −116, −3.5) and FVC (Model 3: β for Q4: −68, 95% CI: −128, −9.0). Trend analyses indicated a statistically significant linear relationship between the quartiles of NHHR and FEV1 (Model 3: p for trend = 0.030) and FVC (Model 3: *p* for trend = 0.015).

**Table 2:** The association between NHHR and lung function parameters.

	NHHR	Model 1 β (95% CI)	Model 2 β (95% CI)	Model 3 β (95% CI)
FEV1				
	Continuous	50 (32, 68)	−31 (−43, −18)	−15 (−28, −2.1)
	Q1	Reference	Reference	Reference
	Q2	20 (−44, 85)	−20 (−59, 20)	−13 (−50, 24)
	Q3	135 (51, 219)	−54 (−103, −4.8)	−32 (−79, 14)
	Q4	178 (93, 263)	−112 (−168, −56)	−60 (−116, −3.5)
	*p* for trend	**<0.001**	**<0.001**	**0.030**
FVC				
	Continuous	74 (56, 93)	−42 (−55, −28)	−21 (−35, −7.2)
	Q1	Reference	Reference	Reference
	Q2	72 (0.33, 143)	−6.6 (−50, 37)	12 (−30, 53)
	Q3	222 (130, 315)	−61 (−116, −6.9)	−18 (−72, 36)
	Q4	285 (197, 374)	−142 (−200, −84)	−68 (−128, −9.0)
	*p* for trend	**<0.001**	**<0.001**	**0.015**
FEV1/FVC				
	Continuous	−1.7 (−3.3, −0.06)	0.67 (−0.48, 1.8)	0.67 (−0.44, 1.8)
	Q1	Reference	Reference	Reference
	Q2	−8.5 (−16, −1.0)	−3.3 (−9.1, 2.5)	−5.2 (−10, −0.10)
	Q3	−8.1 (−15, −1.1)	−0.27 (−5.4, 4.8)	−3.4 (−7.8, 1.1)
	Q4	−9.5 (−16, −2.5)	0.98 (−4.1, 6.1)	−0.37 (−5.2, 4.4)
	*p* for trend	**0.013**	0.5	0.9

Model 1: no covariates were adjusted. Model 2: age, gender and race were adjusted. Model 3: age, gender, race, education level, BMI group, smoking status, physical activity, alcohol, hypertension, PIR, cotinine and diabetes were adjusted. NHHR: Non-High-Density Lipoprotein Cholesterol to High-Density Lipoprotein Cholesterol Ratio, CI: Confidence Interval, FEV1: Forced Expiratory Volume in 1 s, FVC: Forced Vital Capacity, FEV1/FVC: Ratio of Forced Expiratory Volume in 1 s to Forced Vital Capacity. Bold values in the table indicate statistical significance (*p* < 0.05).

### 3.3 Smoothed curve fitting analysis

**Figure 2:**
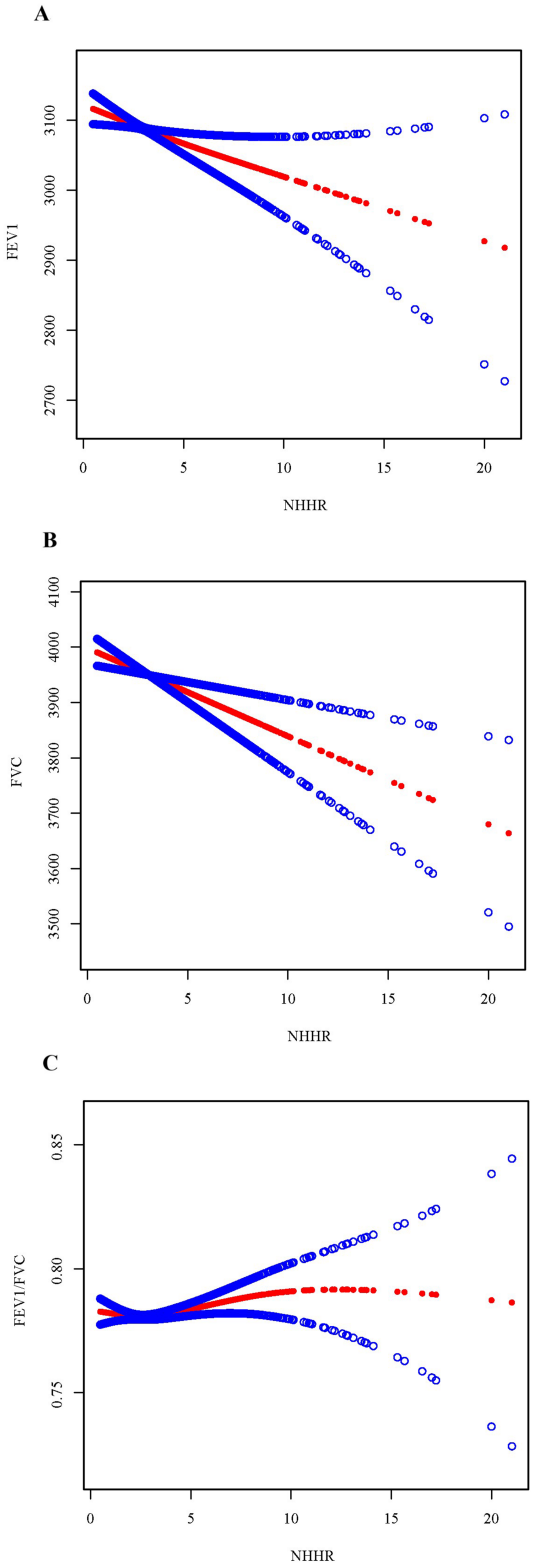
Smoothed curve fitting by generalized additive model between CMI and pulmonary function. **(A)** NHHR and FEV1. **(B)** NHHR and FVC. **(C)** NHHR and FEV1/FVC. NHHR: Non-High-Density Lipoprotein Cholesterol to High-Density Lipoprotein Cholesterol Ratio, FEV1: Forced Expiratory Volume in 1 s, FVC: Forced Vital Capacity, FEV1/FVC: Ratio of Forced Expiratory Volume in 1 s to Forced Vital Capacity.

To further explore and visualize the association between NHHR and pulmonary function, three smoothed curves were generated following the adjustment for all covariates to depict the associations between NHHR and FEV1, FVC, and the FEV1/FVC ratio, respectively (Figure 2). The findings revealed a consistent, linear, and inverse relationship existing between NHHR and both FEV1 and FVC. However, a U-shaped relationship was identified between NHHR and FEV1/FVC (log-likelihood ratio = 0.009). Threshold impact assessment utilizing a two-segment linear regression model (Table 3), an inflection point of 2.3 for the NHHR was identified. When NHHR was below 2.3, a notable inverse relationship with FEV1/FVC was identified (β = −4.8, 95% CI: −9.2, −0.4, *p* = 0.032); however, when NHHR exceeded 2.3, the association turned positive (β = 1.7, 95% CI: 0.5, 2.9, *p* = 0.004).

**Table 3:** Threshold effect analysis of the association between NHHR and lung function parameters.

Pulmonary function		β (95% CI)	p-value
FEV1/FVC	Inflection point	2.3	
	NHHR <2.3	−4.8 (−9.2, −0.4)	**0.032**
	NHHR >2.3	1.7 (0.5, 2.9)	**0.004**
	Log likelihood ratio	**0.009**	

Age, gender, race, education level, BMI group, smoking status, physical activity, alcohol, hypertension, PIR, cotinine and diabetes were adjusted. NHHR: Non-High-Density Lipoprotein Cholesterol to High-Density Lipoprotein Cholesterol Ratio, CI: Confidence Interval, FEV1/FVC: Ratio of Forced Expiratory Volume in 1 s to Forced Vital Capacity. Bold values in the table indicate statistical significance (*p* < 0.05).

### 3.4 Subgroup analysis and interaction testing

**Figure 3:**
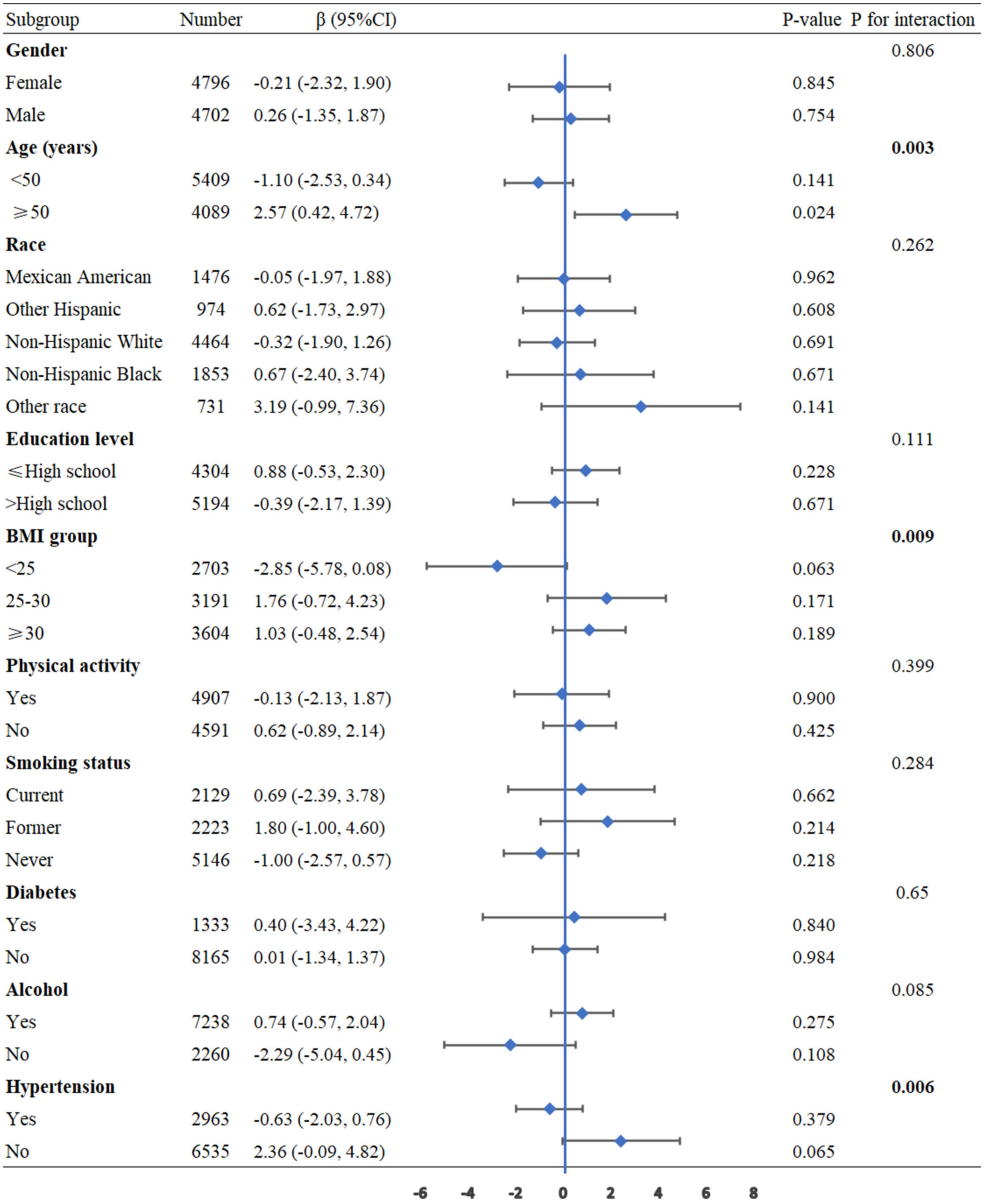
Subgroup analysis between NHHR and the FEV1. BMI: Body Mass Index, CI: Confidence Interval.

**Figure 4:**
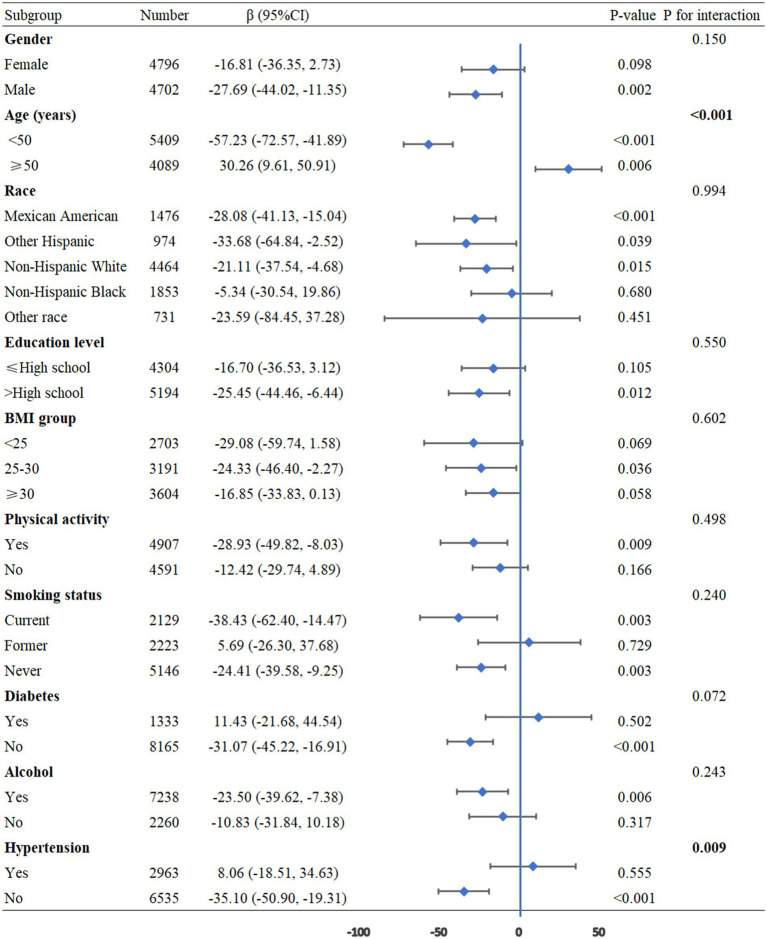
Subgroup analysis between NHHR and the FVC. BMI: Body Mass Index, CI: Confidence Interval.

**Figure 5:**
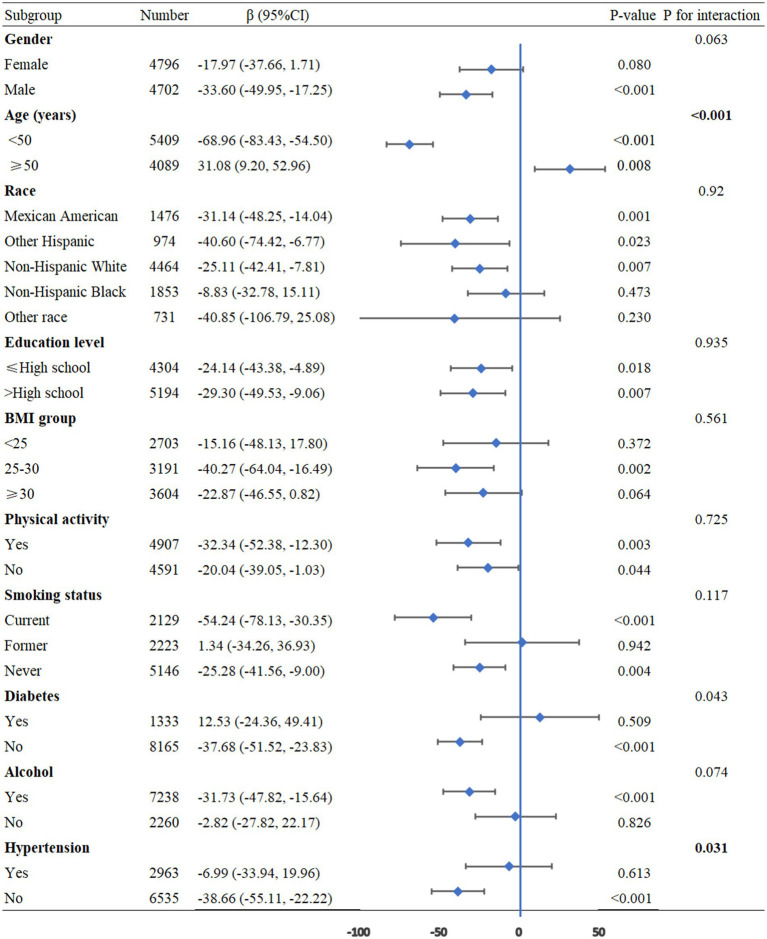
Subgroup analysis between NHHR and the FEV1/FVC. BMI: Body Mass Index, CI: Confidence Interval.

To further assess the robustness and heterogeneity of the association between NHHR and pulmonary function, we performed subgroup analyses categorized by age, gender, race, educational attainment, BMI, physical activity, smoking status, alcohol consumption, diabetes, and hypertension, with adjustments for all confounding factors. The results showed that age (P for interaction <0.001) and hypertension (P for interaction =0.009) had significant interaction effects on the relationship between NHHR and FEV1. Additionally, age (P for interaction <0.001) and hypertension (P for interaction =0.031) exhibited notable interactions in the relationship between NHHR and FVC. For the relationship between NHHR and FEV1/FVC, significant interactions were observed for age (P for interaction = 0.003), BMI (P for interaction = 0.009), and hypertension (P for interaction = 0.006) (Figures 3–5).

### 3.5 Sensitivity analysis

In order to evaluate the stability of the results, a sensitivity analysis was performed by omitting 1,659 participants diagnosed with asthma and chronic obstructive pulmonary disease (COPD). The diagnostic criteria for asthma and COPD are detailed in Supplementary Table S3. The results showed that in Model 2, adjusted for age, gender, and race, NHHR exhibited a substantial negative correlation with FEV1, FVC, and FEV1/FVC. In Model 3, with full adjustment for all covariates, the negative associations between NHHR and FEV1 (β = −21, 95% CI: −34, −7.7) and FVC (β = −29, 95% CI: −43 to −15) persisted, with a significant trend. These findings indicate that the relationship between NHHR and pulmonary function persists as significant even when participants with asthma COPD are excluded from the analysis (Supplementary Table S4).

## 4 Discussion

This research investigated the relationship between the non-HDL-C to HDL-C ratio (NHHR) and PFT among a representative sample of U.S. adults from the NHANES 2007–2012 data. Our results indicate that higher NHHR is significantly associated with lower PFT, specifically FEV1 and FVC, even after adjusting for multiple covariates. This finding suggests that elevated NHHR, an indicator of lipid imbalance, could potentially adversely affect pulmonary function, supporting previous research that links dyslipidemia with respiratory health. The linear negative trend between NHHR and pulmonary function, particularly in the higher quartiles, further highlights the potential role of lipid metabolism in maintaining lung well-being. Interestingly, a U-shaped correlation was identified between NHHR and FEV1/FVC, indicating the complexity of lipid-related effects on lung function. These findings underscore the importance of monitoring lipid profiles in maintaining pulmonary health, especially in populations at risk for dyslipidemia-related conditions.

The findings of this study align with prior studies examining the association between lipid metabolism and lung function in the general adult population. Prior studies have shown that dyslipidemia, particularly low HDL-C levels, is closely related to decreased lung function. For instance, Lee et al. identified a notable association between HDL-C levels and both FEV1 and FVC, with participants having low HDL-C showing notable differences compared to those with normal HDL-C levels. Specifically, individuals with low levels of HDL-C exhibited decreases in FVC and FEV1 by 0.74–2.19% and 0.86–2.68%, respectively (19). Wu et al. further demonstrated a significant association between elevated NHHR and COPD (15). In addition, Wang et al. reported a close relationship between HDL and both the presence and severity of pulmonary hypertension, it is noteworthy that plasma high-density lipoprotein (HDL) levels were found to be significantly reduced in individuals diagnosed with pulmonary hypertension, with HDL serving as an important independent predictor in these patients (20). Similarly, Pan et al. suggested that NHHR could be a potential predictive tool for obstructive sleep apnea, with each incremental increase in NHHR raising the prevalence of obstructive sleep apnea by 9% (21). Our findings extend these observations by showing that NHHR is associated with specific diseases, and closely interrelated to lung function levels among the general public. Furthermore, while previous studies have largely focused on individual lipid markers, our study introduces NHHR as a composite metric, offering a more comprehensive perspective that better captures the impact of lipid metabolism imbalance on lung function. However, research by Wen et al. indicates a negative relevance between HDL levels and pulmonary function within a certain range among COPD patients. When HDL levels fall below 66 mg/dl, high HDL is strongly linked to impaired lung function and gas retention (22). Similarly, Huang et al. confirmed that higher HDL levels correlate with poorer lung function in COPD patients (23). These findings suggest that HDL may influence lung function through different mechanisms depending on the disease status and population. Additional studies are required to investigate the biological processes that contribute to these variations. Notably, in addition to traditional lipid markers, the role of HDL-bound long non-coding RNAs (lncRNAs) in lipid metabolism and vascular health has garnered significant attention. For example, studies have shown that HDL-lncRNA LEXIS is significantly associated with lipoprotein(a) levels and vascular damage as assessed by pulse wave velocity (PWV) (24). This research highlights the role of HDL-bound lncRNAs in the pathophysiology of lipid metabolism disorders, suggesting their potential as novel biomarkers and therapeutic targets in lipid-related diseases. Future studies could explore whether HDL-bound lncRNAs, such as LEXIS, are associated with pulmonary function and whether targeting these molecules could offer new therapeutic strategies for respiratory diseases.

The potential mechanisms by which NHHR affects lung function are not yet fully understood. One possible mechanism involves the inflammatory pathway, where inflammatory responses can lead to airway constriction, airway wall remodeling, and pulmonary fibrosis, all of which contribute to reduced lung function (25). Research has indicated that hypercholesterolemia plays a role in the early stages of interstitial lung injury, inflammation, and fibrosis (26). Hypercholesterolemia is typically characterized by increased levels of non-HDL-C and reduced levels of HDL-C (27). Research by Aihara et al. indicates that patients with chronic interstitial pneumonia/fibrosis exhibit significantly lower HDL-C levels compared to healthy individuals (28). This may be linked to abnormal lipid metabolism that intensifies systemic inflammatory responses, with HDL-C playing a critical role in mitigating lung inflammation and regulating pulmonary surfactant function (29). Moreover, HDL-C exhibits multiple anti-inflammatory functions, this includes the elimination of bacterial toxins, facilitation of corticosteroid secretion, attenuation of platelet aggregation, inhibition of endothelial cell apoptosis, reduction of inflammatory responses in monocytes, and suppressing the expression of endothelial cell adhesion molecules (30). Non-HDL-C encompasses low-density lipoprotein cholesterol (LDL-C), very-low-density lipoprotein cholesterol (VLDL-C), and others, which are significant contributors to atherosclerosis (31), with LDL-C being a primary factor in its development (32). Atherosclerosis is recognized as a chronic systemic inflammatory disorder, frequently associated with elevated levels of reactive oxygen species (ROS) that contribute to oxidative stress (33). Under the combined influence of atherosclerosis and systemic inflammation, pulmonary microvascular damage may occur, impairing gas exchange and lung function. In addition, dyslipidemia may directly affect alveolar structure and airway function. Studies suggest that abnormal blood lipid levels can result in the accumulation of lipids in the lungs, reducing lung elasticity and compliance, which results in decreased lung capacity (manifested as a reduction in FVC) (34). Furthermore, abdominal fat accumulation may restrict diaphragmatic movement, exacerbating lung function decline (35). These mechanisms collectively help explain the observed association between NHHR and decreases in FEV1 and FVC.

Based on our findings, we propose several new hypotheses for future research. First, it is plausible that NHHR may influence lung function through systemic inflammation and oxidative stress pathways. Future studies could explore whether interventions targeting these pathways, such as anti-inflammatory or antioxidant therapies, could mitigate the adverse effects of elevated NHHR on pulmonary function. Second, the U-shaped relationship between NHHR and FEV1/FVC suggests that there may be a threshold effect, where both very low and very high NHHR levels could impair lung function. This hypothesis warrants further investigation to identify the optimal NHHR range for maintaining lung health. Third, the role of lipid-lowering therapies, such as statins, in improving lung function among individuals with elevated NHHR should be explored, particularly in populations at risk for respiratory diseases.

To enhance the clinical relevance of our findings, we propose that NHHR could be integrated into routine clinical assessments as a supplementary tool for evaluating pulmonary health. Given its significant association with reductions in FEV1 and FVC, NHHR could assist physicians in identifying individuals at risk of impaired lung function, particularly those with dyslipidemia or early respiratory symptoms. For example, in primary care settings, an elevated NHHR detected during routine lipid profiling could prompt further evaluation with spirometry, especially in high-risk groups such as smokers or obese patients. Moreover, NHHR might inform therapeutic strategies; patients with high NHHR and concurrent respiratory concerns could benefit from intensified lipid-lowering therapies (e.g., statins), which may reduce systemic inflammation and mitigate lung function decline. While these applications require validation through prospective clinical trials, our study suggests that NHHR could serve as a practical biomarker linking lipid metabolism to pulmonary outcomes, offering a cost-effective approach to early detection and management of respiratory risks.

This study has several notable strengths. First, it utilizes large-scale NHANES 2007–2012 data, ensuring a sample representative of various ages, genders, races, and health statuses, which enhances the generalizability of our findings. Second, we adjusted for multiple confounding variables, encompassing age, gender, race, BMI, and smoking status, and applied strict inclusion and exclusion criteria (e.g., including only A or B quality pulmonary function tests) to improve the robustness and reliability of our results. Additionally, by employing NHHR—a composite lipid metric—rather than single lipid levels (such as LDL-C or HDL-C), this research offers a more thorough understanding of an individual’s lipid metabolic profile.

Nonetheless, this study likewise possesses certain constraints. First, as a cross-sectional study, it lacks the capacity to ascertain causal connections, therefore, the relationship between NHHR and lung function is causal requires further validation through longitudinal studies. Second, despite adjusting for various covariates, residual confounding may still exist, as factors such as dietary habits and physical activity were not fully controlled. Third, self-reported data for variables such as smoking, alcohol consumption, hypertension, diabetes, asthma, and COPD may introduce recall bias or misclassification. Although NHANES uses standardized questionnaires to minimize bias, social desirability bias or inaccurate recall of medical history may still affect the results. Future studies could improve data reliability by incorporating objective measures, such as biomarkers for smoking or medical records. Additionally, NHHR may be influenced by certain medications (e.g., lipid-lowering drugs) or metabolic conditions, which were not fully accounted for in this study. Finally, since this research is grounded in data derived from a U.S. population, further research is needed to determine its applicability to other ethnicities or regions.

## 5 Conclusion

In conclusion, this research demonstrates a notable correlation between the non-HDL-C to HDL-C ratio (NHHR) and pulmonary function, with higher NHHR linked to reductions in key lung function metrics, such as FEV1 and FVC. These findings suggest that lipid metabolic imbalance, as indicated by elevated NHHR, may fulfil a role in lung health beyond traditional cardiovascular implications. The results underscore the importance of considering lipid profiles in assessing respiratory health, especially in individuals at risk for dyslipidemia-related conditions. While our study provides valuable insights, further longitudinal study is a necessary condition for establishing causality and explore the latent mechanisms connecting lipid metabolism to pulmonary function.

## Data Availability

The datasets presented in this study can be found in online repositories. The names of the repository/repositories and accession number(s) can be found at: the NCHS Research Ethics Review Board reviewed and approved the NHANES study protocol. More information can be found on the CDC website at https://www.cdc.gov/nchs/nhanes/irba98.htm.
