# Supplementary material for: The association between non-high-density lipoprotein cholesterol to high-density lipoprotein cholesterol ratio and pulmonary function: evidence from NHANES 2007–2012
Source: Front Nutr. 2025 Mar 19;12:1534958. doi: 10.3389/fnut.2025.1534958 (PMC11961415; doi:10.3389/fnut.2025.1534958)
Supplement: Supplementary file 1 [file Table_1.DOCX]

**Supplementary Table S1.** Detailed information on categorical covariables.

| Covariable | Category | Explanation |
| --- | --- | --- |
| **Race** | Mexican American | Mexican American |
|  | Other Hispanic | Other Hispanic |
|  | Non-Hispanic White | Non-Hispanic White |
|  | Non-Hispanic Black | Non-Hispanic Black |
|  | Other race | Other race |
| **Gender** | Female | Female |
|  | Male | Male |
| **BMI group** | <25 | Normal or underweight |
|  | 25-30 | Overweight |
|  | ≥30 | Obesity |
| **Education level** | ≤High school | Below 11th grade (including 12th grade without a diploma) |
|  | >High school | High school graduate or above GED equivalent |
| **Physical activity** | Yes | Engage in at least 10 minutes of heart rate-boosting activity weekly. |
|  | No | Avoid 10-minute heart rate-boosting activity weekly. |
| **Smoking status** | Never | Smoked fewer than 100 cigarettes in their lifetime. |
|  | Former | Smoked more than 100 cigarettes but is not currently smoking. |
|  | Current | Smoked more than 100 cigarettes and is currently smoking. |
| **Alcohol** | Yes | Consumed at least 12 drinks of any type of alcoholic beverage in the past year. |
|  | No | Consumed fewer than 12 drinks of alcoholic beverages in the past year. |
| **Diabetes** | Yes | HbA1c level ≥6.5%, or fasting blood glucose ≥126 mg/dL, or self-reported doctor diagnosis, or currently using insulin. |
|  | No | Does not meet the above criteria for diabetes diagnosis. |
| **Hypertension** | Yes | Has been informed by a doctor or other healthcare professional that they have high blood pressure. |
|  | No | Has never been informed by a doctor or other healthcare professional that they have high blood pressure. |

| Variable | GVIF | Df | GVIF^(1/(2*Df)) |
| --- | --- | --- | --- |
| NHHR | 1.2126953 | 1 | 1.10122445 |
| Age | 1.46110433 | 1 | 1.20876149 |
| PIR | 1.30180598 | 1 | 1.14096713 |
| Cotinine | 1.87770652 | 1 | 1.37029432 |
| Gender | 1.15804681 | 1 | 1.07612583 |
| Race | 1.30970621 | 4 | 1.0343005 |
| Education level | 1.30346358 | 1 | 1.1416933 |
| BMI group | 1.3006214 | 2 | 1.06791755 |
| Physical activity | 1.15252537 | 1 | 1.07355734 |
| Smoking status | 2.10382258 | 2 | 1.20434878 |
| Diabetes | 1.18625933 | 1 | 1.08915533 |
| Alcohol | 1.18072244 | 1 | 1.08661053 |
| Hypertension | 1.32307997 | 1 | 1.15025213 |

**Supplementary Table S2.** Variance inflation factors for variables.

NHHR, non-HDL-C to HDL-C ratio; GVIF, Generalized Variance Inflation Factor; Df, Degrees of Freedom.


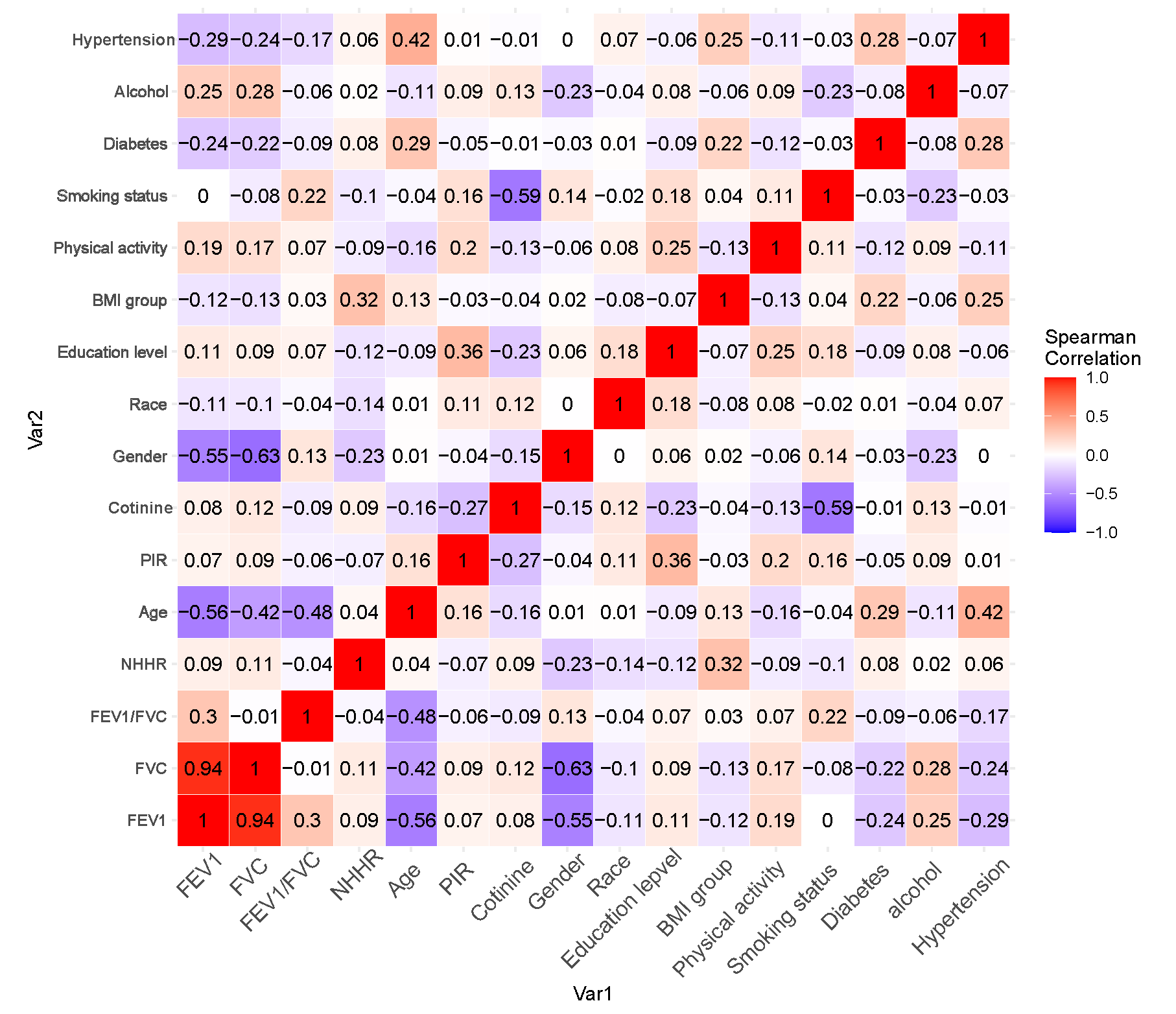


**Supplementary Figure S1.** Heatmap of Spearman’s rank correlation coefficients.

**Supplementary Table S3.** Diagnosis of asthma and COPD in NHANES.

| **Asthma** | Participants were considered to have asthma if they answered "Yes" to the question: "Has a doctor or other health professional ever told you that you have asthma?" |
| --- | --- |
| **COPD** | Participants were considered to have COPD if they met both of the following criteria:1. A self-reported physician diagnosis of chronic bronchitis or emphysema. 2.Spirometry results indicating airflow limitation (FEV1/FVC ratio < 0.70). |

**Supplementary Table S4.** Sensitivity analysis of weighted multiple linear regression models of NHHR with pulmonary function.

|  | NHHR | Model 1  β (95% CI) | Model 2  β (95% CI) | Model 3  β (95% CI) |
| --- | --- | --- | --- | --- |
| FEV1 |  |  |  |  |
|  | Continuous | 55 (38, 71) | −33 (−46, −21) | −21 (−34, −7.7) |
|  | Q1 | Reference | Reference | Reference |
|  | Q2 | 54 (−15, 123) | −20 (−59, 20) | −16 (−56, 23) |
|  | Q3 | 168 (89, 247) | −54 (−103, −9.7) | −42 (−89, 5.2) |
|  | Q4 | 215 (138, 292) | −112 (−165, −59) | −74 (−130, −19) |
|  | *p* for trend | **<0.001** | **<0.001** | **0.006** |
| FVC |  |  |  |  |
|  | Continuous | 80 (63, 97) | −44 (−58, −30) | −29 (−43, −15) |
|  | Q1 | Reference | Reference | Reference |
|  | Q2 | 110 (30, 191) | −11 (−52, 30) | 2.8 (−40, 45) |
|  | Q3 | 262 (171, 353) | −66 (−119, −12) | −34 (−90, 23) |
|  | Q4 | 325 (241, 409) | −144 (−201, −87) | −91 (−152, −30) |
|  | *p* for trend | **<0.001** | **<0.001** | **0.001** |
| FEV1/FVC |  |  |  |  |
|  | Continuous | −1.7 (−3.2, −0.26) | 0.47 (−0.69, 1.6) | 0.43 (−0.77, 1.6) |
|  | Q1 | Reference | Reference | Reference |
|  | Q2 | −7.5 (−15, −0.42) | −2.1 (−7.9, 3.6) | −4.2 (−9.5, 1.2) |
|  | Q3 | −8.5 (−16, −1.0) | −0.59 (−6.4, 5.3) | −3.6 (−8.9, 1.7) |
|  | Q4 | −8.6 (−15, −2.3) | 1.1 (−4.0, 6.2) | −0.12 (−5.2, 5.0) |
|  | *p* for trend | **0.012** | 0.6 | >0.9 |

Model 1: no covariates were adjusted. Model 2: age, gender and race were adjusted. Model 3: age, gender, race, education level, BMI group, smoking status, physical activity, alcohol, hypertension, PIR, cotinine and diabetes were adjusted.
